# 439. Corowa-kun: Impact of a COVID-19 Vaccine Information Chatbot on Vaccine Hesitancy, Japan 2021

**DOI:** 10.1093/ofid/ofab466.638

**Published:** 2021-12-04

**Authors:** Takaaki Kobayashi, Yuka Nishina, Hana Tomoi, Ko Harada, Eiyu Matsumoto, Kanako Inaba, Jun Ishihara, Shugo Sasaki, Kenta Horimukai, Kyosuke Seguchi, Kyuto Tanaka, Hiromizu Takahashi, Jorge L Salinas, Yuji Yamada

**Affiliations:** 1 University of Iowa Hospitals and Clinics, Iowa city, Iowa; 2 Department of General Medicine Juntendo University Faculty of Medicine, Bunkyo, Tokyo, Japan; 3 London School of Hygiene and Tropical Medicine, London, England, United Kingdom; 4 Okayama University Graduate School of Medicine, Dentistry and Pharmaceutical Sciences, Okayama, Okayama, Japan; 5 University of Iowa, Iowa City, Iowa; 6 Kanto Central Hospital, Minato-ku, Tokyo, Japan; 7 Imperial College London, London, England, United Kingdom; 8 Saitama Medical University Hospital, Kawagoe, Saitama, Japan; 9 Jikei University Katsushika Medical Center, Katsushika-ku, Tokyo, Japan; 10 Kameda Medical Center, Kamogawa, Chiba, Japan; 11 Kawasaki Municipal Hospital, Kawasaki, Kanagawa, Japan; 12 Juntendo University Fuculty of Medicine, Chiyoda, Tokyo, Japan; 13 University of Iowa Hosptials and Clinics, Iowa City, IA; 14 Icahn School of Medicine at Mount Sinai, New York, New York

## Abstract

**Background:**

Japan has one of the highest vaccine hesitancy rates in the world. According to a previous study, less than 30% of people strongly agreed that vaccines were safe, important, or effective. We created a COVID-19 vaccine information chatbot in a popular messenger app in Japan to answer COVID-19 vaccine frequently asked questions (FAQs) via text messages. We assessed the impact of chatbot text messages on COVID-19 vaccine hesitancy by conducting a cross-sectional survey among chatbot users.

**Methods:**

LINE is the most popular messenger app in Japan; about 86 million people in Japan (roughly two-thirds of the population) use this messenger app. Corowa-kun, a free chatbot, was created in LINE on February 6, 2021. Corowa-kun provides instant, automated answers to frequently asked COVID-19 vaccine questions. A cross-sectional survey assessing COVID-19 vaccine hesitancy was conducted via Corowa-kun during April 5 to 12, 2021. We included persons ages 16 years old and older who had not received a COVID-19 vaccine. The survey was written in Japanese and consisted of 21 questions.

Corowa-kun’s Consultation Room

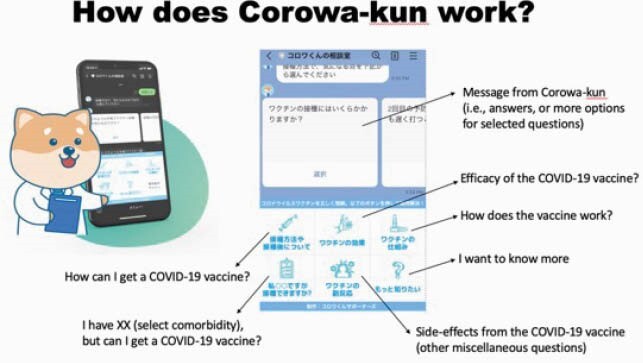

Corowa-kun is the mascot of an online chatbot. This chatbot in LINE is used to answer COVID-19 vaccine frequently asked questions (FAQs) via text messages. As of May 10th, 70 FAQs are available.

**Results:**

A total of 59,676 persons used Corowa-kun during February to April 2021. The most commonly accessed message categories were: “I have (select comorbidity), can I get a COVID-19 vaccine?” (23%); followed by questions on adverse reactions (22%) and how the vaccine works (20%). 10,192 users (17%) participated in the survey. Median age was 55 years (range 16 to 97), and most were female (74%). Intention to receive a COVID-19 vaccine increased from 59% to 80% after using Corowa-kun (p < 0.01). Overall, 20% remained hesitant: 16% (1,675) were unsure, and 4% (364) did not intend to be vaccinated. Factors associated with vaccine hesitancy were: age 16 to 34 (odds ratio [OR] = 3.7, 95% confidential interval [CI]: 3.0–4.6, compared to age ≥65), female sex (OR = 2.4, Cl: 2.1–2.8), and history of another vaccine side-effect (OR = 2.5, Cl: 2.2–2.9). Being a physician (OR = 0.2, Cl: 0.1-0.4) and having received a flu vaccine the prior season (OR = 0.4, Cl: 0.3-0.4) were protective.

COVID-19 vaccine acceptance increased and hesitancy decreased after using Corowa-kun, Japan, 2021 (n=10,192)

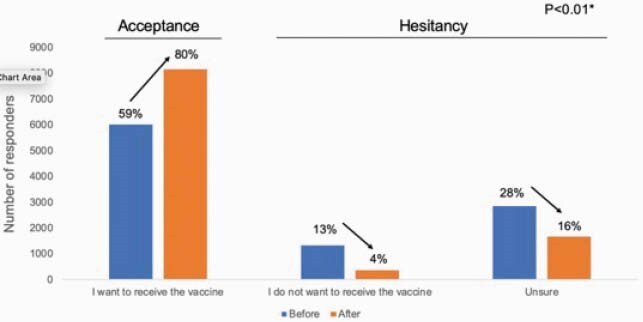

*There was a statistically significant difference in responses between before and after using Corowa-kun (p < 0.01, Chi-square test).

Univariable logistic regression models of factors associated with COVID-19 vaccine hesitancy, Japan, 2021 (n=10,192)

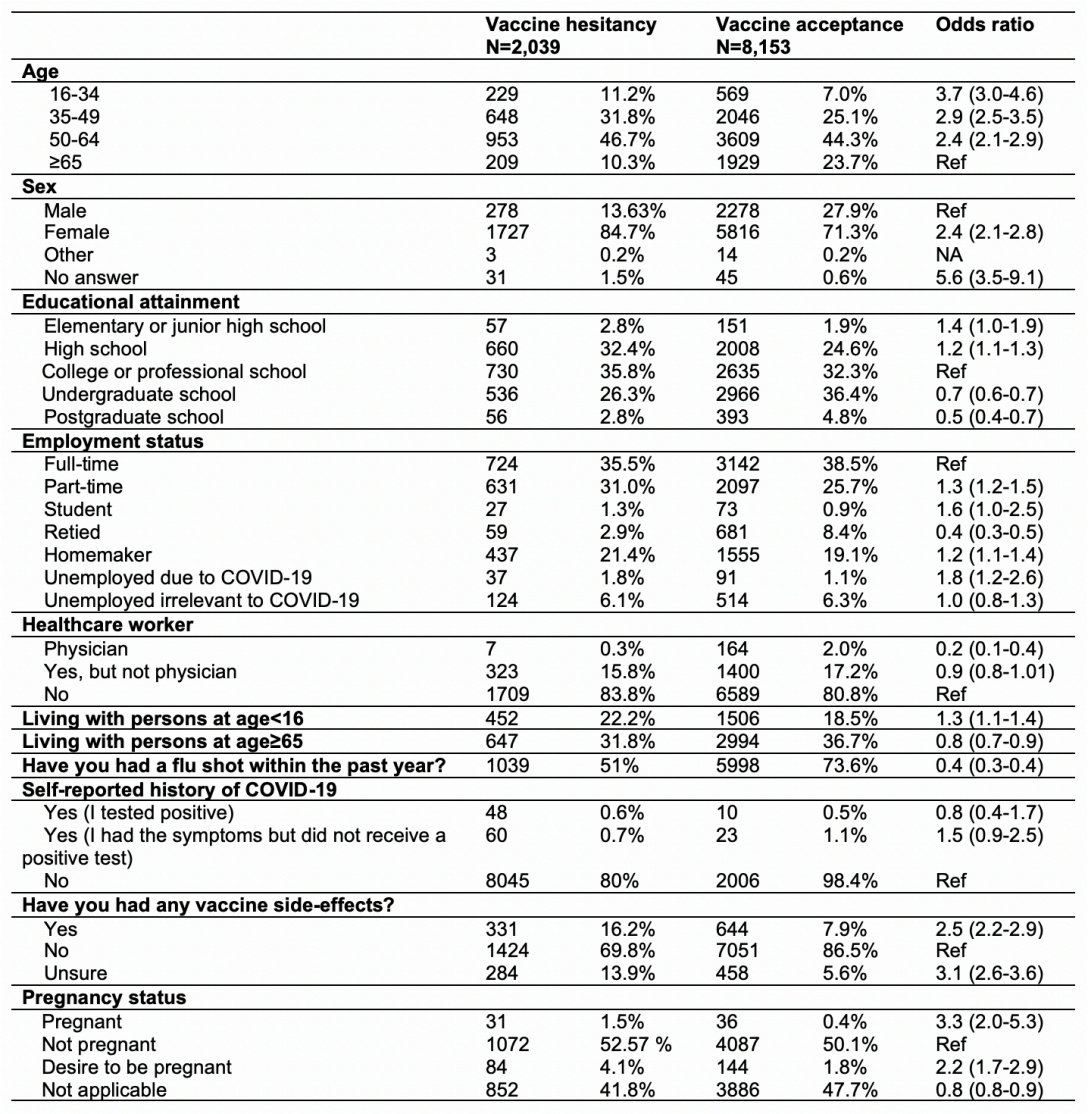

Ref: reference NA: Logistic regression was not performed due to too small number (n≤3)

**Conclusion:**

Corowa-kun reduced vaccine hesitancy by providing COVID-19 vaccine information in a messenger app. Mobile messenger apps could be leveraged to increase COVID-19 vaccine acceptance.

**Disclosures:**

**All Authors**: No reported disclosures

